# Laparoscopic Banded One Anastomosis Gastric Bypass: A Single-Center Series

**DOI:** 10.1155/2022/4942052

**Published:** 2022-01-29

**Authors:** Michela Campanelli, Emanuela Bianciardi, Domenico Benavoli, Giulia Bagaglini, Giorgio Lisi, Paolo Gentileschi

**Affiliations:** ^1^Department of Bariatric and Metabolic Surgery, San Carlo of Nancy Hospital and University of Rome “Tor Vergata”, Rome, Italy; ^2^Chair of Psychiatry, Department of Systems Medicine, University of Rome “Tor Vergata”, Rome, Italy; ^3^General Surgery Residency School, University of Rome “Tor Vergata”, Rome, Italy; ^4^Department of Surgery, Sant'Eugenio Hospital, Viale Dell'Umanesimo 10, Rome 00144, Italy

## Abstract

**Introduction:**

Laparoscopic one anastomosis gastric bypass (LOAGB) is a relatively new procedure for the treatment of morbid obesity and related comorbidities. On average, this procedure results in good postoperative weight loss with a low complication rate. Recent publications suggest that dumping syndrome and weight regain might be reduced by placing a silicone ring over the gastric pouch during the procedure, so called laparoscopic banded one anastomosis gastric bypass (LBOAGB).

**Methods:**

86 patients undergoing LBOAGB between 2018 and 2020 were enrolled in this retrospective study. Hospital records were used to assess weight loss, comorbidity resolution, and any complications either in the short or medium term.

**Results:**

54 Female and 32 male patients were included with a mean age of 43 years (25–64), preoperative body mass index of 42 kg/m^2^ (35–49), and preoperative weight of 114 kg (86–162). Thirty-four patients presented with type 2 diabetes (39.5%), 42 patients (49%) diagnosed with hypertension, 24 presented with OSAS (28%), and 21 (24%) hypercholesterolaemia patients were included. In total, 36 patients were diagnosed with multiple comorbidities. The operative data showed an average operative time of 48 minutes with 3.4% of patients suffering from early (minor) complications and 2.3% with a late (minor) complication. One patient required reoperation due to intra-abdominal bleeding. The median length of hospital stay was 2.5 days. Median follow-up was 18 months (5–36). In that period, no patient required ring removal or conversion to Roux-en-Y gastric bypass surgery. Food intolerance/vomiting was present in 1 patient (1.1%), bile reflux was present in 1 patient (1.1%), and no stomal ulcers were observed. Mean % excess weight loss at 12 and 24 months was 72% and 80%, respectively. Fifty-two out of 86 patients (60%) had a complete resolution of comorbidities. A CONUT score >2 (mild malnutrition) was found in 40% of patients, while a CONUT score 0-1 was found in 60% of patients.

**Conclusion:**

LBOAGB shows promising results in terms of safety and efficacy in the short term. Further prospective studies will be required to evaluate the consistency of the results in the long term.

## 1. Introduction

Bariatric surgery provides a wide range of weight loss procedures as effective treatment for morbid obesity and obesity-related comorbidities. Each procedure is associated with varying degrees of weight loss accomplishment, comorbidity resolution, and perioperative or long-term complications. The procedure best suitable for each individual patient is determined by a combination of the patients' body mass index (BMI), eating behaviours, and comorbidities and the surgeons' experience [1–6].

These surgical procedures can improve esophageal motility and lead to esophageal complications such as gastroesophageal reflux disease (GERD) [7].

The objective of bariatric surgery is to lose excess weight and its related health problems, with minimum risk to the patient, and achieving a good quality of life, while maintaining reduced weight years after surgery [8, 9]. As for other digestive procedures, laparoscopic surgery has determined in bariatric operations a minimally invasive postoperative period and a decrease of postoperative complications [10].

Laparoscopic one anastomosis gastric bypass (OAGB-MGB) was presented for the first time around the turn of the century for the treatment of morbid obesity and its related comorbidities in bariatric surgery [11, 12]. Rutledge was the first to define it as a modification of Mason's loop gastric bypass. [13, 14]. The OAGB-MGB in simple terms is a loop anastomosis of the ilium attached to a long and vertical lesser curvature-based gastric pouch.

The LOAGB is nowadays considered a safe and effective operation and is recognised by most international scientific bariatric federations. The resolution of comorbidities, the postoperative excess weight loss (EWL), and quality of life are significant while the rate of complications is extremely low [15–21], while still providing the chance of revision and conversion when required [22]. Although its outcomes are, in general, good, weight regain remains a significant concern; weight loss may diminish by pouch dilation. One other phenomenon is dumping syndrome in OAGB-MGB patients that reduces the patient`s quality of life and even increases nutrient intake in many.

Recent works suggest that weight regain along with dumping syndrome might be limited by the placement of a silicone ring around the gastric pouch during the procedure [23, 24]. The size of the ring is adjustable with a locking mechanism during the procedure.

As for laparoscopic sleeve gastrectomy (LSG) and the laparoscopic Roux-en-Y gastric bypass (LRYGB), weight loss associated with silicone band placement is due to the synergism of malabsorptive and restrictive effect: limiting food intake, promoting early satiety, altering gastric emptying, hormonal pathways, secretion, and peristalsis [25–28]. This technique reduces the gastric pouch dilation preventing weight regain.

Although there are many publications on primary and secondary silicone ring placements in LSG and LRYGB patients, they are lacking for OAGB-MGB patients. The aim of this study was to report the short-term outcomes of a cohort of patients undergoing primary laparoscopic banded one anastomosis gastric bypass (LBOAGB).

## 2. Materials and Methods

The study analysed prospectively collected data of a clinical trial. Between January 2018 and October 2020, 86 patients undergoing surgery for morbid obesity were selected for LBOAGB. A total of 1624 bariatric procedures were performed during the study period. Patients were selected to banded OAGB-MGB according to age, BMI, eating disorders, and comorbidities.

A minimizer ring was chosen based on our abundant experience with banded sleeves. Exclusion criteria included patients under the age of 18 or over the age of 65, with major mental health illness, pregnancy, and major medical conditions contraindicating surgery. All patients were informed in detail about the risks and the benefits of the procedure. All the participants gave their written informed consent. Hospital records, follow-up visits, and telemedicine were used to assess weight loss, comorbidity resolution, and complications.

### 2.1. Preoperative Evaluation

Preoperative evaluation was performed by a multidisciplinary team consisting of a bariatric surgeon, specialised bariatric nurse, psychiatrist, anaesthesiologist, cardiologist, endocrinologist, and a nutritionist. The preoperative assessment and interview aimed to explore the patient`s dieting history, preoperative weight, comorbidities, and expectations and eagerness regarding bariatric surgery [29, 30].

Every single patient was evaluated for esophagitis, gastroesophageal reflux, bile reflux, and HP. infection. Each patient was, consequently, submitted to a diagnostic endoscopy, ph-manometry, and biopsy. GERD patients were excluded and submitted to RYGB.

### 2.2. Surgical Technique

In the laparoscopic technique after the establishment of the pneumoperitoneum and the placement of laparoscopic ports, the stomach is divided using linear staples around a 28–36 Fr orogastric tube at the body-antrum junction creating a lesser curvature gastric tube. A gastroenterostomy is performed, between the tubular stomach and the jejunal loop at 200 cm distal to the ligament of Treitz pulled up antecolic. Different surgeons may use different limb lengths. All the procedures were performed laparoscopically, using five ports. A vertical, isolated gastric pouch was created using staples (Medtronic, Johnson) starting below the crow's foot and up to the left crus. An afferent, biliopancreatic limb of 200 cm was created.

After the creation of a retrogastric tunnel starting from the pars flaccida of the hepatogastric ligament, the ring was placed approximately 4 cm distal from the cardia hiatus, around the distal third of the gastric pouch a few centimetres above the gastrojejunostomy.

A prefabricated, radiopaque Minimizer Ring (Bariatric Solutions International GmbH, Switzerland) was placed and closed at the 7 cm closing position in all patients and rhen sutured onto the stomach with two nonabsorbable materials using the suture loops on the device.

### 2.3. Postoperative Management

Patients were evaluated at 1 week, 1 month, 6 months, 1 year after surgery, and annually thereafter for 3 years.

Postoperative advice involved a diet including purees and clear liquids for the first 20 postoperative days followed by semisolid food for the next 10 days. Solid diet started one month after surgery after an additional dietary and nutritional consultation.

Follow-up together with telemedicine and hospital records was used to assess postoperative weight loss, comorbidity resolution, complications, other operations, or hospital admissions.

### 2.4. Data Analysis

Preoperative patients' data such as demographics, BMI, weight, pharmacological therapy, and comorbidity data (both obesity and nonobesity related) were recorded prospectively.

Intraoperative and postoperative collected data included operative time, adverse events or complications, hospital stay, and weight loss. The nutritional status of LBOAGB patients in the postoperative period has been calculated with the CONUT score [31].

A comprehensive scoring system, controlling nutritional status (CONUT), consisting of serum albumin, cholesterol levels, and lymphocyte count in peripheral blood, was used to evaluate patient nutritional status.

The primary outcome of the study was to evaluate the weight loss, analysing data after a median follow-up of 18 months. Secondary outcomes were the assessment of complications and the resolution of comorbidities.

## 3. Results

Baseline patient demographics are reported in Table 1. Eighty-six patients underwent surgery with LBOAGB (54 females, 32 males) with a mean age of 43 (25–64), BMI of 42 kg/m^2^ (35–49), and preoperative weight of 114 kg (86–162). Preoperative comorbidities were reported by or diagnosed in 61 patients (70%). Specifically, 34 patients presented with diabetes (39.5%), 42 patients (49%) reported hypertension, 24 presented with OSAS (28%), and 21 (24%) hypercholesterolaemia with 36 patients presenting multiple comorbidities.

### 3.1. Operative Outcomes

The average operative time skin to skin was 48 minutes. All procedures were performed laparoscopically without any conversions. The median length of hospital stay was 2.5 days. Median follow-up was 18 months (5–36). Mean % EWL at 12 and 24 months was 72% and 80%, respectively (Table 2). Fifty-two out of 86 patients (60%) had a complete resolution of comorbidities. Three patients (3.4%) suffered from early minor complications (Clavien–Dindo I–IIIa): one reported vomiting/food intolerance (grade I), and 2 suffered from wound infection (grade I). Two patients (2.3%) suffered from late minor complications: one experienced bile reflux (grade I), and one reported vomiting/food intolerance (grade I).

Surgical reintervention was needed in one patient for postoperative bleeding from the port insertion site (Clavien–Dindo IIIb). Among patients, no one required either ring removal or conversion to a Roux-en-Y gastric bypass (RYGBP). No one presented with an anastomotic leak. Food intolerance/vomiting was present in 1 patient (1.1%), and bile reflux was present in 1 patient (1.1%); no stomal ulcers were observed. In our series of LBOAGB, a CONUT score >2 (mild malnutrition) was found in 40% of patients, while a CONUT score 0-1 was found in 60% of patients. There were no deaths.

## 4. Discussion

Within the term “banded procedures,” specific bariatric operations are included (with the exclusion of adjustable gastric banding which is based solely on the positioning of an inflatable band around the stomach) where a ring or a band is added to a traditional procedure (RYGB, LSC, and others) to prevent long-term pouch dilation. In recent years, banded bariatric procedures have shown to influence weight loss and weight regain years after surgery [32–36].

Banded operations are not free of postoperative and long-term complications which influence the surgeons' mind creating a bias in their clinical management of morbidly obese patients. As a matter of fact, long-term band-related complications are a challenge for the surgeon and the endoscopist and often require open surgery to be resolved [37, 38].

Nevertheless, the incidence of band-related complications after standard bariatric operations (apart from gastric banding as mentioned above) is low [32, 39, 40]. They are best treated in specialised institutions where the experience with banded procedures is high and the availability of operative endoscopy, interventional radiology, and laparoscopic techniques can help manage these cases [37, 41]. The most popular banded procedure is by far the RYGBs [36, 42]. Several studies have reported better results with banded RYGB in terms of weight loss, weight maintenance, and decreased weight regain compared to the traditional counterpart [40, 42–44]. The main concept is based on the prevention of dilation of the gastric pouch rather than in the increased restriction. In other words, surgeons are not looking for an augmented restriction, which could increase the chance of a leak, but for a tool which prevents or decreases the long-term natural dilation of the pouch, responsible for weight regain [42, 43]. For banded RYGB, a good level of evidence is provided for its dominance over standard gastric bypass when evaluating meta-analyses and RCTs [43, 44].

In recent years, some authors started to adopt the concept of “preventing dilation” and sleeve gastrectomy, and various reports appeared in the literature in the last decade [32, 45, 46]. In our centre, there is considerable experience with banded sleeve gastrectomy [27]. An earlier published RCT from our centre demonstrated that the ring prevents pouch dilation and decreases the incidence of weight regain in the midterm, up to four years after surgery [47].

As for Banded One Anastomosis Gastric Bypass (LBOAGB), only few reports are available in literature and is, either only anecdotical or includes few patients [23, 34, 35, 48]. To our knowledge, only one other good series of patients treated with silastic ring mini gastric bypass and a long-term follow-up of 11 years has been published to date [24]. In this study, a total of 156 patients were submitted to laparoscopic banded OAGB with a good mean percentage excess weight loss %EWL of 84.3% at 11 years. Unfortunately, 3.6% of patients needed ring removal and 9.4% required conversion to RYGB. In addition, the number of patients on antireflux medications increased from 5.1% to 44.6% at 11 years. The authors concluded that banded OAGB is a safe and effective procedure; however, concerns about bile reflux appeared to be well founded. Our series is smaller (86 patients), and the follow-up is shorter (up to 36 months). On the other hand, our patients experienced a satisfactory weight loss, there were no postoperative complications, and no ring was removed. Furthermore, bile reflux was diagnosed only in one patient and no conversion to RYGB was needed. The reason why we are not seeing postoperative ring complications might be the short follow-up of our study even if we believe that a proper preoperative selection of patients is crucial in avoiding side effects and complications [49].

The idea and potential benefit of banded one anastomosis gastric bypass are based on various hypotheses. The first is the prevention of dilation as for RYGB or sleeve. One anastomosis gastric bypass is, basically, a malabsorptive procedure, but restriction plays a role, mainly in the first year. Some degree of weight regain is reported, and it could be related to increased eating attitudes in the long term [50, 51].

Adding a ring may decrease the rate of long-term dilation, as observed for RYGB. Second, the ring might improve collateral and bothersome effects of the procedure such as bile reflux and dumping syndrome. It might also mitigate important side effects such as diarrhoea and, therefore, improve anal symptoms which frequently occur in LOAGB patients [52–54].

In our series, we compared the nutritional status of LBOAGB patients with a cohort of LOAGB patients without finding significant differences between the two groups. Hence, the positioning of a ring has no effects on the malnutrition related to OAGB.

We acknowledge a main limitation of this study based on the short postoperative follow-up even if the rate of patients lost to it is extremely low (2, 3%).

Whether the ring may play a significant role in preventing reflux, dumping episodes, and diarrhoea should be a matter of investigation in physiological studies. In our series, reflux was limited and no stomal ulcers were observed.

## 5. Conclusions

Laparoscopic BOAGB is a safe and efficacious procedure with excellent preliminary short-term results. Weight loss in the first year is durable, and dumping syndrome and bile reflux are limited. Complication rate in the LBOAGB is comparable to that in the traditional LOAGB. In case of complications, the ring can be easily removed laparoscopically. Further studies will be required to evaluate the consistency of the results also in the long term.

## Figures and Tables

**Table 1:** Baseline patient demographics.

Characteristic	
Age y-o (mean/range)	43 (25–64)
Sex (M/F)	32/54
Preop weight (mean/range)	114 (86–162)
Preop BMI (mean/range)	42 (35–49)
Preop comorbidities	86
Hypertension	42
Type 2 diabetes	34
Hypercholesterolemia	21
OSAS	24

Y-o: years old, BMI: body mass index (kg/m^2^), OSAS: obstructive sleep apnea syndrome.

**Table 2:** Postoperative outcomes.

Postoperative data	
Average operative time (min)	36
Median length of hospital stay (days)	2.5
Median follow-up (mts, range)	18 (5–36)
Mean % excess weight loss at 12 months	72%
Mean % excess weight loss at 24 months	80%
CONUT score 0-1	60%
CONUT score >2	40%

MIN, minutes; MTS, months.

## Data Availability

The data used to support the findings of this study are included within the article.
